# A miniature inverted-repeat transposable element, AddIn-MITE, located inside a WD40 gene is conserved in Andropogoneae grasses

**DOI:** 10.7717/peerj.6080

**Published:** 2019-01-11

**Authors:** Clicia Grativol, Flavia Thiebaut, Sara Sangi, Patricia Montessoro, Walaci da Silva Santos, Adriana S. Hemerly, Paulo C.G. Ferreira

**Affiliations:** 1 Laboratório de Química e Função de Proteínas e Peptídeos/Centro de Biociências e Biotecnologia, Universidade Estadual do Norte Fluminense, Campos dos Goytacazes, Rio de Janeiro, Brazil; 2 Laboratório de Biologia Molecular de Plantas/Instituto de Bioquímica Médica Leopoldo De Meis, Universidade Federal do Rio de Janeiro, Rio de Janeiro, Rio de Janeiro, Brazil

**Keywords:** MITEs, Genic region, Small RNAs, Sugarcane, Maize, Sorghum, Andropogoneae, WD40 gene

## Abstract

Miniature inverted-repeat transposable elements (MITEs) have been associated with genic regions in plant genomes and may play important roles in the regulation of nearby genes via recruitment of small RNAs (sRNA) to the MITEs loci. We identified eight families of MITEs in the sugarcane genome assembly with MITE-Hunter pipeline. These sequences were found to be upstream, downstream or inserted into 67 genic regions in the genome. The position of the most abundant MITE (Stowaway-like) in genic regions, which we call AddIn-MITE, was confirmed in a WD40 gene. The analysis of four monocot species showed conservation of the AddIn-MITE sequence, with a large number of copies in their genomes. We also investigated the conservation of the AddIn-MITE’ position in the WD40 genes from sorghum, maize and, in sugarcane cultivars and wild *Saccharum* species. In all analyzed plants, AddIn-MITE has located in WD40 intronic region. Furthermore, the role of AddIn-MITE-related sRNA in WD40 genic region was investigated. We found sRNAs preferentially mapped to the AddIn-MITE than to other regions in the WD40 gene in sugarcane. In addition, the analysis of the small RNA distribution patterns in the WD40 gene and the structure of AddIn-MITE, suggests that the MITE region is a proto-miRNA locus in sugarcane. Together, these data provide insights into the AddIn-MITE role in Andropogoneae grasses.

## Introduction

Transposable elements (TEs) have important roles in plant genome evolution due to the high efficient copy number increase through a copy-and-paste and cut-and-paste mechanisms (Bennetzen, Ma & Devos, 2005; Bennetzen, 2007). Since their first description as mobile elements by Barbara McClintock, TEs have been correlated with changes in chromosome structure and gene expression patterns (McClintock, 1950). TEs jumping into genomic regions can create novel genetic variation by insertions into coding regions or by perturbing gene regulatory networks through insertions into gene control regions (Feschotte, Jiang & Wessler, 2002). Class I TE movement involves an intermediate RNA and a copy-and-paste mechanism. While class II TEs movement is mediated by a transposase that recognizes TIR (terminal inverted repeats) sequences. Miniature inverted-repeat transposable elements (MITEs) are a special class of TEs with a structure similar to class II transposons, but without encoding transposase (Casacuberta & Santiago, 2003).

MITEs are amplified to high copy numbers in the genome of various plants such as rice, with more than 90,000 MITES grouped into 100 different families (Feschotte & Wessler, 2002). In rice and Arabidopsis, most of the MITEs are inserted into euchromatic (Wright, Agrawal & Bureau, 2003), instead of heterochromatic regions, which seem to have important roles in the emergence of phenotypic diversity (Lu et al., 2012). Since sugarcane does not yet have a complete published genome, the examination of repetitive content has been performed on BAC assemblies and also on the assembled sugarcane transcripts (Vettore et al., 2003). The analysis of BAC sequences showed that MITEs represent only 3% of repetitive sequences, compared with 40% of LTR retrotransposons sequences (De Setta et al., 2014). Araujo and collaborators (Araujo et al.) found 267 transcripts with similarity to TEs from closely related species. The TE, known as *Mutator*, showed expression in almost all analyzed sugarcane tissues. Interestingly, the authors reported that the expression of different TEs was drastically induced in sugarcane callus, which was also reported on Arabidopsis callus (Tanurdzic et al., 2008). In addition, Grativol et al. (2014) found MITEs frequently distributed among methyl filtered genomic sugarcane sequences, suggesting that this group is less methylated in sugarcane in comparison to other TEs. More recently, the monoploid genome assembly using a minimum tiling path approach showed that TEs represent 52% of the assembled R570 cultivar genome (Garsmeur et al., 2018).

The close physical association of MITEs and plant genes (Mao et al., 2000) suggests that MITEs could play a role in gene regulation. Studies have shown that in rice, MITEs affect the expression of nearby genes (Lu et al., 2012). In the Solanaceae family, they down-regulate gene expression through MITE-derived small RNAs (Kuang et al., 2009). An important step to advance the knowledge of TEs function was the discovery that the TEs’ activity, and therefore their mobility, can be suppressed by epigenetic mechanisms via RNA-dependent DNA methylation (RdDM) and histone modifications (Law & Jacobsen, 2010; Ng, Lu & Chen, 2012). Silencing of TEs has been mostly attributed to a class of small RNAs, the siRNAs (small interfering RNAs) (Malone & Hannon, 2009). The siRNAs are the most abundant sRNA class and are predominantly produced from transposable elements, heterochromatic regions or other repetitive sequences (Xie et al., 2004; Obbard & Finnegan, 2008). The functional role of siRNAs is to direct DNA methylation in genomic loci from where they were originated and silence resident TEs in *cis* (Law & Jacobsen, 2010). It has also been reported that siRNAs pathways may influence the transcription of neighbor protein-coding genes as well as modify the epigenetic status of upstream sequences (Wang et al., 2011).

Sternes & Moyle (2014) first reported the correlation of MITEs, genes, and sRNAs in sugarcane, which showed the generation of sRNA sequences from a MITE located at an intronic region of the PYRUVATE ORTHOPHOSPHATE DIKINASE gene (POPDK). Ortiz-Morea et al. (2013) and Zanca et al. (2010) also reported small RNA sequences derived from active MITEs in sugarcane genome. The discovery that in humans, Arabidopsis and rice several families of miRNAs, another class of small RNAs, are derived from MITEs (Piriyapongsa, Mariño Ramírez & Jordan, 2007; Piriyapongsa & Jordan, 2008) supports the evidence that MITEs can play an important evolutionary role silencing genes, not only in plants but also in other eukaryotes. However, the impact of MITEs, particularly when associated with plant genes, remains poorly explored.

Here, we have used the MITE-Hunter pipeline for identification of MITEs in a genomic assembly of methyl-filtered sequences from sugarcane (Grativol et al., 2014). The association of identified MITEs and sugarcane genes were found. One Stowaway-like MITE (AddIn-MITE) was found in WD40 genic regions from sorghum, maize, and sugarcane. Then, the AddIn-MITEs were evaluated for their conservation in different sugarcane wild species and commercial cultivars, as well as close related monocots species. Finally, the role of AddIn-MITE as a sRNA producer at a sugarcane gene possibly involved in plant stress responses was investigated.

## Material and methods

### MITE identification

The identification of MITE families in 674 Mb of sugarcane genomic assembly (Grativol et al., 2014) was performed through a computational approach for *de novo* identification of these elements - MITE-Hunter pipeline (Han & Wessler, 2010). The pipeline of the MITE-Hunter has four main steps: (i) identification of MITE candidates; (ii) filter out of false positives through a pairwise sequence alignment; (iii) selection of MITE examples; (iv) filter out of false positives through a multiple sequence alignment; (v) grouping of predicted MITE consensus sequences into families.

The MITE-Hunter was run with default parameters with the sugarcane genome sequence file (sugarcaneMFscaffolds-1.0.fa.gz) downloaded from http://lbmp.bioqmed.ufrj.br/genome/download_sequence. The predicted sugarcane MITE sequences were manually inspected for the presence of TSD (target site duplications). Only MITEs with identified TSDs were selected for further analysis. The fasta format sequences of identified MITEs are available at Data S1.

### Identification and annotation of genes associated with MITEs in sugarcane

In total, 145 sugarcane scaffolds containing MITE sequences with TSDs were aligned against *Sorghum bicolor* version 3.1 coding sequences (CDS) by BLASTN (Altschul et al., 1990) with the *E*-value cutoff of 10^−10^. Only the best hits were considered for identification of genes nearby MITEs. The sorghum CDSs and their annotation were downloaded from JGI Phytozome 12.

To confirm the annotation of exons regions in the scaffold5050—size5174 containing the gene with a WD40 domain, we used the raw sugarcane RNA-seq reads of four control samples (Vargas et al., 2014; Santa Brigida et al., 2016) downloaded from NCBI SRA under accessions (SRX374577, SRX374581, SRX603441 and SRX603445). The quality of the libraries was evaluated using FASTX Toolkit (http://hannonlab.cshl.edu/fastx_toolkit/) and reads having base quality greater or equal to 20 (Q20) was used for additional analyzes. Filtered RNA-seq reads were mapped using Bowtie2/2.1.0 (Langmead & Salzberg, 2012) onto the WD40 gene with default settings.

### WD40 annotation and phylogenetic tree

For the construction of the phylogenetic tree, WD40 protein sequences from sorghum and maize were obtained in Phytozome 12. We used the annotation file of sorghum and maize genes to select only those genes annotated with WD-40 or WD repeat domain. This WD-40 domain (PF00400) was confirmed in all protein sequences from selected genes using Pfam (https://pfam.xfam.org/). Additionally, we performed a BLASTN search on a sugarcane transcriptome (TR7) (Santa Brigida et al., 2016) using the sugarcane Scaffold5050 annotated as WD40 protein. One transcript from the Locus_26251 was selected. The protein sequence predicted from this sugarcane transcript were obtained from Expasy translate tool. All selected protein sequences were aligned using MUSCLE (Edgar, 2004).

The evolutionary history was inferred by using the Maximum Likelihood method based on the Whelan and Goldman model (Wheland & Goldman, 2001). Initial tree(s) for the heuristic search were obtained automatically by applying Neighbor-Join and BioNJ algorithms to a matrix of pairwise distances estimated using estimated using a JTT model approach, and then selecting the topology with superior log-likelihood value. The rate variation model allowed for some sites to be evolutionarily invariable ([+I ], 3,37% sites). The analysis was performed by 1,000 generations and a consensus tree was generated to assign an a posteriori probability to each node using the 1,000 trees sampled. The tree was drawn to scale, with branch lengths measured in the number of substitutions per site. The analysis involved 12 amino acid sequences. All positions containing gaps and missing data were eliminated. There were a total of 274 positions in the final dataset. Evolutionary analyses were conducted in MEGA7 (Kumar, Stecher & Tamura, 2016).

The gene structure and synteny analysis of Sobic.001G093800 and GRMZM2G056645 were performed at Gene Structure Display Server v2.0 and Plant Genome Duplication Database (http://chibba.agtec.uga.edu/duplication/), respectively.

### Validation of AddIn-MITE position at sugarcane genome

Following the Kuijper’s leaf numbering system for sugarcane plants (Cheavegatti-Gianotto et al., 2011), leaf -2 tissue of each sugarcane wild species and cultivars were collected for DNA extraction. Young leaves were collected from plants of *S. Spontaneum* clone SES205A, *S. officinarum* clone 82–72, *S. robustum* clone Molokai5009, *S. barberi* clone Khagziand, *S. sinense* clone Chukche maintained in the germplasm collection of Instituto Agronômico de Campinas, Ribeirão Preto and Monsanto Breeding Station, Maceió. Samples from SP70-1143 and other cultivars were collected from our labs’ collection. Total genomic DNA was extracted from leaves using the CTAB method (Doyle & Doyle, 1987) with minor modifications. The quality and concentration of DNA were estimated using Thermo Scientific *NanoDrop*™ 2000c Spectrophotometer and then the integrity was verified by electrophoresis on a 1% agarose gel.

PCR amplification was performed using 5ng of DNA from each sample. Specific primers for the validation of MITEs location were designed using the Primer3 program and are listed in Table S1. The amplification reactions were performed with a final volume of 25 µL. In each reaction, 50 mM KCl, 10 mM Tris-HCl (pH 8.0), 1 mM MgCl2, 0.2 mm dNTPs (Promega^®^, Madison, WI, USA), 1U Taq DNA polymerase (New England Biolabs) and 200 nM of the primer were used. The PCR program started with an initial denaturation at 94 °C for 5 min followed by 12 touchdown cycles of 30 s at 94 °C; 45 s of annealing temperature, starting at 52 °C and decreasing 0.5 °C per cycle; and 2 min at 72 °C. Another 30 cycles with annealing temperature fixed at 46 °C and 10 min at 72 °C were used as a final step. Amplified products were observed by electrophoresis in 0.5 × TAE at 100 V using 2% agarose gel with a 100-bp ladder as standard (Promega).

### Conservation analysis of sugarcane AddIn-MITE

Estimation of AddIn-MITE copy number and conservation among monocots’ genomes were performed through TARGeT: Tree Analysis of Related Genes and Transposons (Han, Burnette & Wessler, 2009). The BLASTN tool was run with sugarcane MITE sequences against *Brachypodium dystachyon*, *Oryza sativa*, *Panicum virgatum*, *Setaria italica*, *Sorghum bicolor* and *Zea mays* genomes. The coverage of the sugarcane AddIn-MITE and number of hits on each genome were plotted. To test the co-localization of the AddIn-MITE with genes in the various monocots genomes, the AddIn-MITE sequence was used as the query on NCBI/BLASTN against non-redundant (nr) sequences. All genes containing an AddIn-MITE inserted in genic regions or flanking sequences were selected and the location of the AddIn-MITE was analyzed.

### Analysis of small-RNAs derived from AddIn-MITE

Small RNA libraries from leaves of sugarcane (GSM1040783), sorghum (GSM803128) and maize (GSM433620) were downloaded from NCBI’s Gene Expression Omnibus. The sRNA reads from each species were aligned against the MF scaffolds from sugarcane and the WD40 genic region associated with AddIn-MITE from maize and sorghum, using the sequence alignment tool from UEA sRNA toolkit-Plant Standalone version. The alignments were performed with non-redundant sRNAs, zero mismatches allowed and visualized at the same toolkit. Next, the aligned sRNAs were distributed by size. The hairpin structures of AddIn-MITE on the three species were obtained on RNAFold Web Server.

To measure the abundance of AddIn-MITE-derived sRNAs generated in sugarcane plants subject to pathogen infection and salt stress, the sRNA libraries under accessions codes GSM1350704, GSM1350705, GSM1040783, and GSM1040786 were downloaded from NCBI’s Gene Expression Omnibus. The alignments of WD40 genic region and sRNAs were performed as described above. The venn diagram was constructed with the sRNAs sequences mapped on AddIn-MITE region.

## Results

### Identification of MITEs in sugarcane genomic assembly

**Figure 1:**
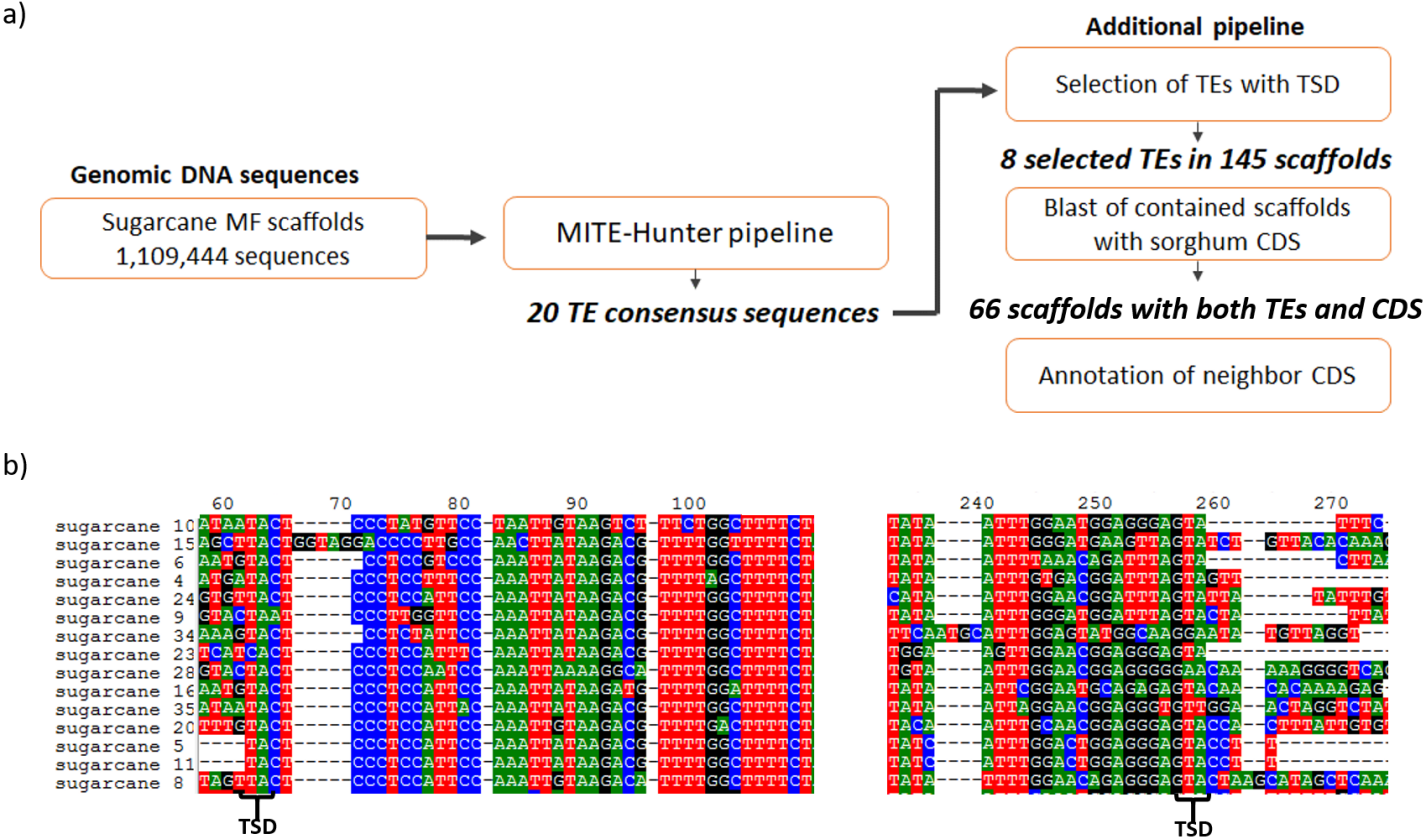
Identification of MITEs in sugarcane. (A) The pipeline used to identify MITEs in sugarcane. MITE-Hunter program was used to identify MITE candidate from sugarcane methyl filtrated (MF) scaffolds. An additional step was performed to select TEs with TSD regions, which have 2–10 bp. The 145 scaffolds containing MITEs with TSDs were aligned against sorghum CDSs. (B) Example of sugarcane MITEs identified by this pipeline, highlighting the presence of 2 bp TSD in the boundaries of the MITE.

The search for MITEs in the sugarcane genomic assembly of methyl-filtered reads (MF scaffolds) was performed following the MITE-Hunter pipeline, which was run using 1,109,444 sugarcane MF scaffolds (Grativol et al., 2014). Five major steps constitute the pipeline and were used to obtain 20 TE consensus sequences (Fig. 1A). An additional pipeline was applied to select TEs with TIR-like structures flanked by TSDs, which have 2–10 bp. After that, eight valid TE exemplars were selected from the 20 TE consensus sequences, which distributed among 145 sugarcane MF scaffolds (Table 1). The most common TSDs size was 3 bp for each candidate. Figure 1B shows the presence of 2 bp TSDs in the boundaries of one of the identified MITEs. The size of identified ranged from 106 to 155 bp. The sequences of sugarcane MITEs were compared with the structures of plant MITEs already described in the literature. We found that sugarcane_1_16642 has a similar structure of Stowaway MITE, as described by Bureau & Wessler (1994). Six sugarcane MITEs were similar to Tourist superfamily (Bureau & Wessler, 1992; Zhang et al., 2004).

**Table 1:** MITEs families identified in sugarcane genome with their TSD sequence and number of scaffolds that contain MITEs.

**MITE_ID**	**TSD**	**Size (bp)**	**Scaffolds containing MITEs**	**Superfamily**
sugarcane_1_16642	2 bp	147	34	Stowaway-like
sugarcane_2_15424	3 bp	138	35	Tourist-like
sugarcane_1_4411	3 bp	139	35	Tourist-like
sugarcane_3_10739	3 bp	123	10	Tourist-like
sugarcane_5_2717	3 bp	126	7	Tourist-like
sugarcane_1_2650	4 bp	155	13	n.d.
sugarcane_3_6227	3 bp	106	8	Tourist-like
sugarcane_1_19766	3 bp	126	3	Tourist-like

**Notes.**

n.d. non detected

### Gene-associated MITEs

Evidence that MITEs could be associated with genic regions in the sugarcane genome were indicated by the low methylation level of this class of transposon when compared to retrotransposons (Grativol et al., 2014). In the Arabidopsis genome, MITEs are distributed throughout euchromatic regions (Wright, Agrawal & Bureau, 2003). Based on this, we have searched for genes located in neighboring regions of sugarcane MITEs. The 145 scaffolds containing eight distinct MITE families with TSDs were submitted to BLASTN against sorghum CDS to predict exonic regions on sugarcane genomic sequences. Sixty-six scaffolds showed significant alignments with sorghum CDSs (Fig. 1A). Detailed analysis of MITE insertions through the start-end position of sorghum CDSs showed that the majority of them are localized at intronic regions of genes. Only three MITEs are localized upstream and five are downstream of the CDSs (Table 2). MITEs were located in genes involved with plant development (e.g., WD40-REPEAT FAMILY PROTEIN, REPLICATION FACTOR C1 and VACUOLELESS1); hormone response (e.g., AUXIN-RESPONSIVE PROTEIN IAA1); cell wall formation (e.g., CALLOSE SYNTHASE 8 and FASCICLIN-LIKE ARABINOGALACTAN PROTEIN 17); stress response (e.g., CALCIUM-DEPENDENT PROTEIN KINASE 4, LIPID-TRANSFER PROTEIN 1, TOBAMOVIRUS MULTIPLICATION PROTEIN 3 and VASCULAR PLANT ONE ZINC FINGER PROTEIN); and epigenetic modifications (e.g., HAC13 HISTONE ACETYLTRANSFERASE and O-METHYLTRANSFERASE FAMILY 2 PROTEIN) (Table 2). The MITE family with greater copy number within genic regions of sugarcane was the sugarcane_1_16642, which we call AddIn-MITE (a piece of sequence that can be added to a region to give extra features or functions) (Table 2).

**Table 2:** Summary of sixty-six sugarcane scaffolds containing MITEs and CDS. The gene annotation and the MITE location are also shown.

**MITE**	**Sugarcane scaffold**	**Sorghum CDS**	**Gene annotation**	**MITE localization^a^**
sugarcane_1_19766	scaffold1216—size7184	Sobic.010G115200.1	Ribosomal protein L7Ae/L30e/S12e/Gadd45 family protein	Inside
sugarcane_1_19766	scaffold108051—size2067	Sobic.010G016100.1	Malectin/receptor-like protein kinase family protein	Downstream
sugarcane_1_16642	scaffold5050—size5174	Sobic.001G093800.1	Beige/BEACH domain;WD40 domain, G-beta repeat protein	Inside
sugarcane_1_16642	scaffold6441—size4101	Sobic.006G091300.1	Thioredoxin family protein	Inside
sugarcane_1_16642	scaffold20509—size3611	Sobic.005G160100.1	replication factor C1	Inside
sugarcane_1_16642	scaffold28724—size2333	Sobic.004G098000.2	haloacid dehalogenase-like hydrolase family protein	Inside
sugarcane_1_16642	scaffold53770—size2094	Sobic.001G431700.1	Cytochrome c oxidase biogenesis protein Cmc1-like	Downstream
sugarcane_1_16642	scaffold56570—size2092	Sobic.004G107800.1	CALS8 (CALLOSE SYNTHASE 8)	Inside
sugarcane_1_16642	scaffold57609—size2091	Sobic.008G053300.1	calcium-dependent protein kinase 4	Inside
sugarcane_1_16642	scaffold58260—size2091	Sobic.009G081800.2	RAD3-like DNA-binding helicase protein	Upstream
sugarcane_1_16642	scaffold64421—size2087	Sobic.010G169900.1	Protein phosphatase 2A, regulatory subunit PR55	Inside
sugarcane_1_16642	scaffold99995—size2072	Sobic.001G313500.1	tobamovirus multiplication protein 3	Inside
sugarcane_1_16642	scaffold128323—size1533	Sobic.006G278200.1	RNA-binding (RRM/RBD/RNP motifs) family protein	Inside
sugarcane_1_16642	scaffold134519—size1244	Sobic.002G068100.1	DDT domain superfamily	Inside
sugarcane_1_16642	scaffold147805—size977	Sobic.003G059750.1	WD40/YVTN repeat-like-containing domain;Bromodomain	Inside
sugarcane_1_16642	scaffold156024—size888	Sobic.003G264200.1	flower-specific, phytochrome-associated protein phosphatase 3	Inside
sugarcane_1_16642	scaffold157439—size875	Sobic.010G115200.1	Ribosomal protein L7Ae/L30e/S12e/Gadd45 family protein	Inside
sugarcane_1_16642	scaffold164188—size822	Sobic.004G087600.1	RNA-binding (RRM/RBD/RNP motifs) family protein	Inside
sugarcane_1_16642	scaffold171946—size773	Sobic.004G145000.2	SF35 - IMPORTIN-7, 8, 11	Inside
sugarcane_1_16642	scaffold177647—size743	Sobic.003G217300.2	RING/FYVE/PHD zinc finger superfamily protein	Inside
sugarcane_1_16642	scaffold181625—size725	Sobic.001G439300.1	S-adenosyl-L-methionine-dependent methyltransferases superfamily protein	Inside
sugarcane_1_16642	scaffold187902—size698	Sobic.003G401600.1	THO2	Inside
sugarcane_1_16642	scaffold223651—size590	Sobic.002G330600.1	eukaryotic translation initiation factor-related	Inside
sugarcane_1_16642	scaffold228218—size579	Sobic.002G119400.1	Protein kinase superfamily protein	Inside
sugarcane_1_16642	scaffold271005—size504	Sobic.001G428900.1	S-locus lectin protein kinase family protein	Inside
sugarcane_1_16642	scaffold613983—size292	Sobic.010G160300.1	CTC-interacting domain 11	Inside
sugarcane_1_16642	scaffold851273—size236	Sobic.001G428900.1	S-locus lectin protein kinase family protein	Inside
sugarcane_1_16642	scaffold898319—size228	Sobic.001G137800.1	ankyrin repeat family protein	Inside
sugarcane_1_16642	scaffold1095170—size201	Sobic.001G428900.1	S-locus lectin protein kinase family protein	Inside
sugarcane_1_2650	scaffold9692—size3968	Sobic.002G335600.4	eukaryotic translation initiation factor 4G	Inside
sugarcane_1_2650	scaffold20326—size3628	Sobic.009G082700.1	S-locus lectin protein kinase family protein	Inside
sugarcane_1_2650	scaffold23720—size2977	Sobic.006G129700.1	Flavin containing amine oxidoreductase family	Upstream
sugarcane_1_2650	scaffold27588—size2395	Sobic.001G256400.3	SLOW GROWTH 1	Dowstream
sugarcane_1_2650	scaffold123175—size2037	Sobic.003G294100.1	pyrimidin 4	Inside
sugarcane_1_2650	scaffold139685—size1109	Sobic.008G030900.1	lipid transfer protein 1	Inside
sugarcane_1_2650	scaffold589261—size299	Sobic.004G265600.1	Tetratricopeptide repeat (TPR)-like superfamily protein	Inside
sugarcane_1_4411	scaffold27400—size2406	Sobic.008G103700.1	SPIRAL1-like1	Inside
sugarcane_1_4411	scaffold29415—size2303	Sobic.006G024000.1	UDP-glucosyltransferase 74F2	Inside
sugarcane_1_4411	scaffold62185—size2089	Sobic.007G038800.1	Erythronate-4-phosphate dehydrogenase family protein	Dowstream
sugarcane_1_4411	scaffold91442—size2076	Sobic.001G265600.1	Galactose mutarotase-like superfamily protein	Inside
sugarcane_1_4411	scaffold130193—size1418	Sobic.002G308300.1	Unknown protein	Inside
sugarcane_1_4411	scaffold134709—size1238	Sobic.007G023800.1	P-loop containing nucleoside triphosphate hydrolases superfamily protein	Inside
sugarcane_1_4411	scaffold140271—size1098	Sobic.002G154400.1	P-loop containing nucleoside triphosphate hydrolases superfamily protein	Inside
sugarcane_1_4411	scaffold161467—size842	Sobic.007G038700.1	RING/U-box superfamily protein	Inside
sugarcane_1_4411	scaffold245483—size545	Sobic.010G059700.1	Galactosyltransferase family protein	Inside
sugarcane_1_4411	scaffold253437—size531	Sobic.001G187000.2	AMP-dependent synthetase and ligase family protein	Upstream
sugarcane_1_4411	scaffold342030—size426	Sobic.003G072000.3	Leucine-rich repeat protein kinase family protein	Inside
sugarcane_1_4411	scaffold498488—size333	Sobic.001G130100.1	Unknown protein	Inside
sugarcane_1_4411	scaffold794101—size247	Sobic.010G206900.1	HAC13 protein (HAC13)	Inside
sugarcane_2_15424	scaffold6898—size4046	Sobic.010G180600.1	indole-3-acetic acid inducible 14	Inside
sugarcane_2_15424	scaffold8259—size3990	Sobic.002G406900.1	OsFBX444 - F-box domain containing protein, expressed	Inside
sugarcane_2_15424	scaffold21159—size3547	Sobic.009G198000.1	O-methyltransferase family protein	Inside
sugarcane_2_15424	scaffold25314—size2628	Sobic.001G092500.1	Protein kinase superfamily protein	Inside
sugarcane_2_15424	scaffold126254—size1723	Sobic.001G063500.1	FASCICLIN-like arabinogalactan protein 17 precursor	Inside
sugarcane_2_15424	scaffold132138—size1330	Sobic.002G123000.1	3\′-5\′ exonuclease domain-containing protein	Inside
sugarcane_2_15424	scaffold137393—size1160	Sobic.003G298100.1	vascular plant one zinc finger protein	Inside
sugarcane_2_15424	scaffold151129—size937	Sobic.004G101800.1	PEBP (phosphatidylethanolamine-binding protein) family protein	Inside
sugarcane_2_15424	scaffold202854—size645	Sobic.001G290500.1	BR-signaling kinase 2	Inside
sugarcane_2_15424	scaffold643782—size283	Sobic.005G007900.1	Protein phosphatase 2C family protein	Inside
sugarcane_2_15424	scaffold1095847—size201	Sobic.006G237000.1	3-ketoacyl-acyl carrier protein synthase III	Inside
sugarcane_3_10739	scaffold60177—size2090	Sobic.001G480000.1	F-box/RNI-like/FBD-like domains-containing protein	Dowstream
sugarcane_3_10739	scaffold119367—size2051	Sobic.009G227800.1	MAC/Perforin domain-containing protein	Inside
sugarcane_3_10739	scaffold312644—size453	Sobic.008G150800.1	receptor like protein 45	Inside
sugarcane_3_6227	scaffold32041—size2236	Sobic.003G156500.1	Transducin/WD40 repeat-like superfamily protein	Inside
sugarcane_3_6227	scaffold183782—size715	Sobic.003G249600.1	vacuoleless1 (VCL1)	Inside
sugarcane_5_2717	scaffold130220—size1417	Sobic.010G125000.1	steroid nuclear receptor, ligand-binding, putative, expressed	Inside

**Notes.**

a Position of MITEs related to the CDSs. Inside –intronic or exonic position.

**Figure 2:**
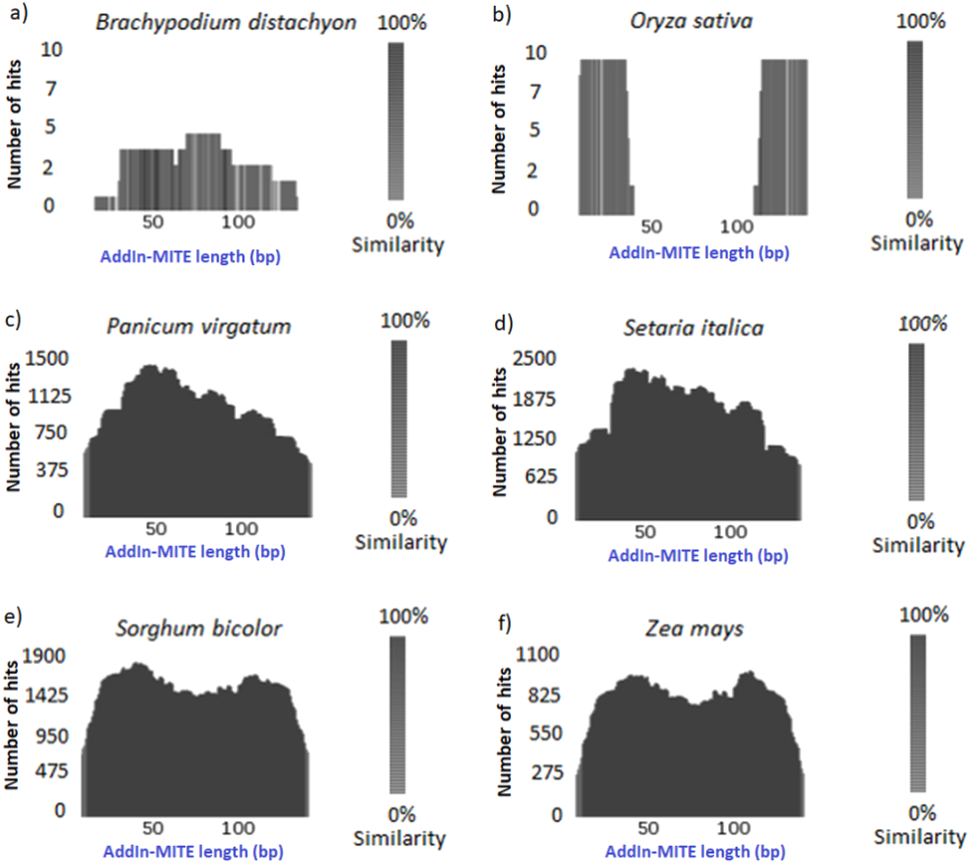
Conservation of the AddIn-MITE sequence. (A) *Brachypodium distachyon*, (B) *Oryza sativa*, (C) *Panicum virgotum*, (D) *Setaria italica*, (E) *Sorghum bicolor*, (F) *Zea mays*. Estimation of AddIn-MITE copy number and conservation among monocots’ genomes were performed through TARGeT. The BLASTN tool was run with sugarcane AddIn-MITE sequences against *Brachypodium distachyon*, *Oryza sativa*, *Panicum virgatum*, *Setaria italica*, *Sorghum bicolor* and *Zea mays* genomes. The coverage of the sugarcane AddIn-MITE and number of hits on each genome were plotted.

Once we found that the AddIn-MITE was associated with sugarcane genic regions, we extended the analysis to other monocot genomes. First, the conservation of the AddIn-MITE sequence in the other six-monocot genomes was analyzed through TARGeT. The analysis of the number of hits and the coverage showed that the AddIn-MITE is conserved in closely related species of Panicoideae sub-family, such as *Panicum virgatum*, *Setaria italica*, *Sorghum bicolor* and *Zea mays* (Fig. 2). In addition, BLASTN of the AddIn-MITE sequence against the non-redundant database showed that this MITE is associated with genic regions also in these four genomes (Table S2). In *Z. mays*, *S. bicolor*, *S. italica* and *S. viridis*, AddIn-MITE have similar localization inside genes. The analysis of AddIn-MITE position on those genes revealed that it is located in intronic regions (Table S2). However, no conservation was observed in species outside the Panicoideae sub-family such as *Oryza sativa* and *Brachypodium distachyon* (Figs. 2A, 2B)

### AddIn-MITE conserved position in WD40 genes of sugarcane, sorghum, and maize

**Figure 3:**
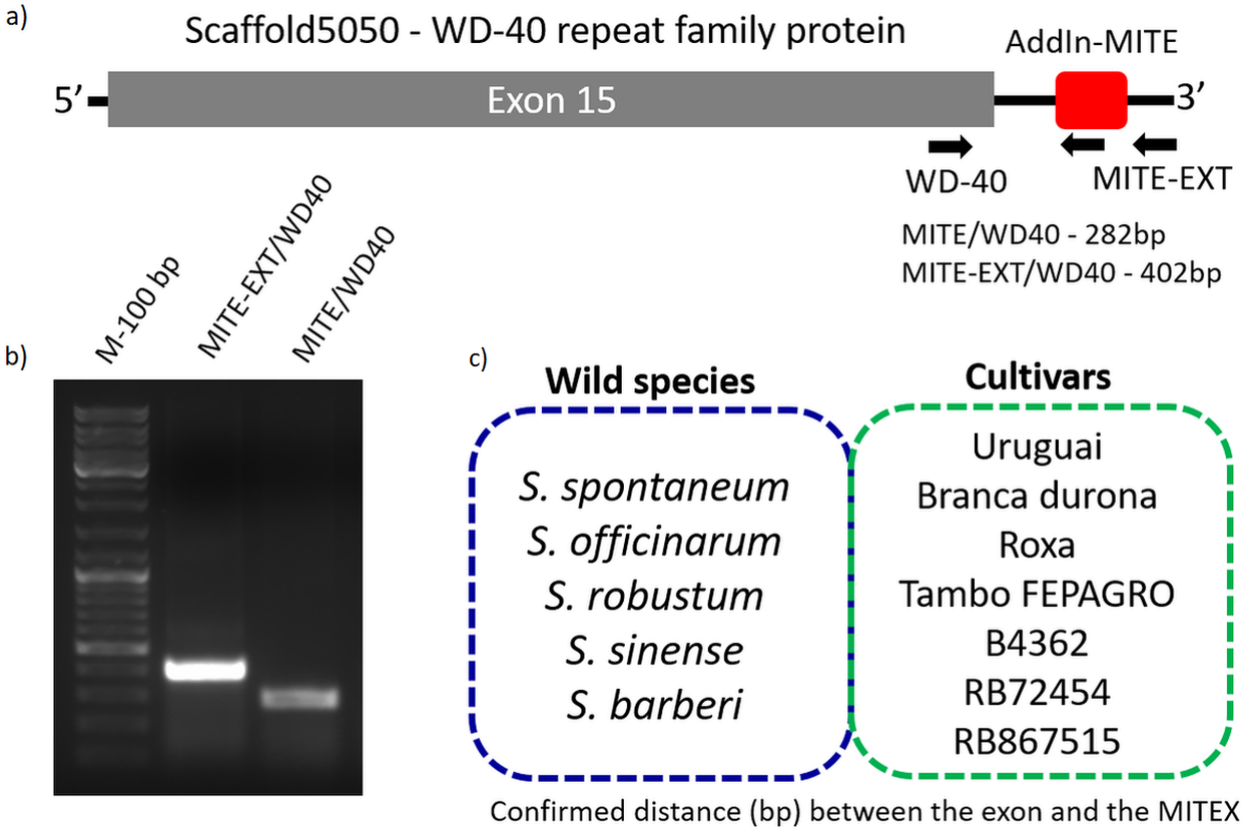
Validation of AddIn-MITE’s position nearby gene. Sugarcane_1_16642 (AddIn-MITE) was the most abundant MITE associated with sugarcane genes. (A) The scheme illustrated that primers were designed inside the AddIn-MITE; inside the CDS and outside the both AddIn-MITE and CDS regions (black arrows). The expected sizes for the combinations of primers were also showed on the figure. (B) PCR reaction with gDNA from the SP70-1143 sugarcane cultivar showed the distance between the AddIn-MITE and the WD40 exon region. M-100 bp ladder was used to confirm the length of the PCR products. (C) In the blue and green boxes are listed the sugarcane wild species and cultivars which the distance (bp) between the exon and the AddIn-MITE was confirmed.

Because sugarcane commercial cultivars have complex, aneuploidy genome, with a history of several rounds of crossings (Grivet & Arruda, 2002), we investigated whether the AddIn-MITE location nearby WD40 gene was conserved in *Saccharum* wild species and hybrids. Figure 3A shows the location of the AddIn-MITE close to the WD40 exon 14 at the scaffold5050 and the position where the primers were designed for PCR. Three primers were designed: (i) one inside the AddIn-MITE; (ii) one inside the CDS and (iii) one outside both the AddIn-MITE and CDS regions (Fig. 3A). PCR reactions with gDNA from the SP70-1143 cultivar validated the distance among the AddIn-MITE and CDS region in the scaffold (Fig. 3B).

The position of AddIn-MITE nearby the WD40 exon was also verified in five wild sugarcane species and seven hybrid cultivars (Fig. 3C). All wild species and cultivars showed the amplified products with expected sizes for the distance between AddIn-MITE and WD40 exon (Fig. S1). Additionally, we confirmed the position of the AddIn-MITE inside the CALS8 gene in the wild species and cultivars (Fig. S2).

**Figure 4:**
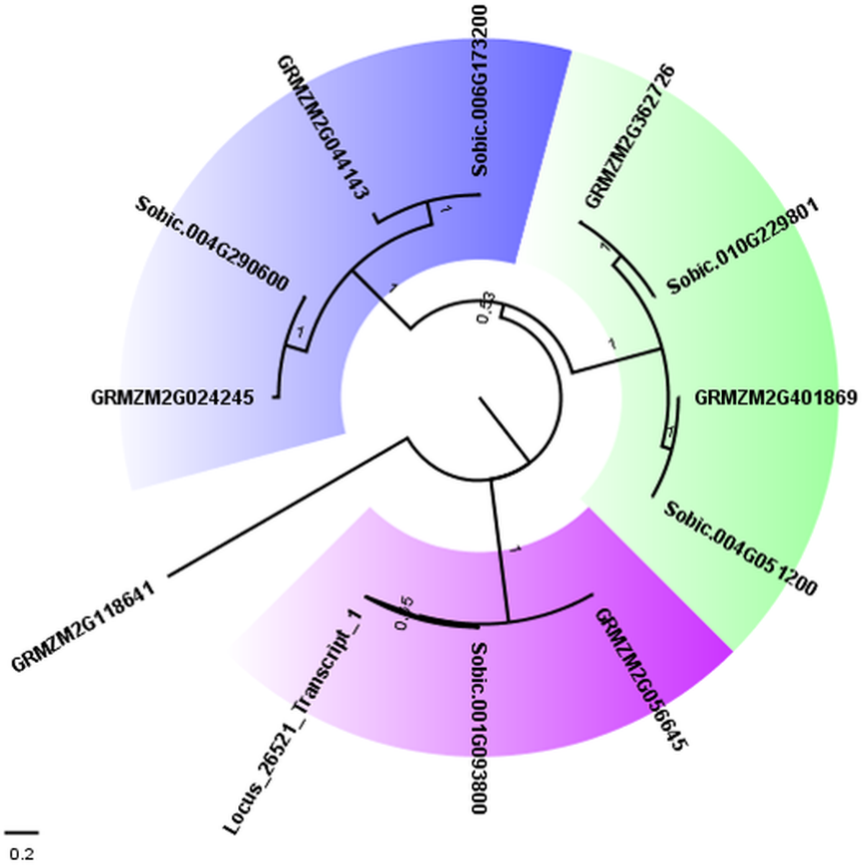
Phylogenetic tree of WD40 proteins from sugarcane, sorghum, and maize. The maximum likelihood phylogenetic analysis of residues from the WD40 proteins from subfamily C was performed using the Whelan and Goldman model with invariable sites. Parameters estimated from 1,000 trees were used to calculate a posterior probability at each node and to generate a consensus tree. The subgroups of proteins were marked with different colors in the tree. Bootstrap values ranged from 0.53 to 1.0.

To confirm the close relationship between AddIn-MITE and genes, we analyzed the WD40 gene annotated on the sugarcane scaffold5050 (Table 2). First, we selected all proteins from the largest transcripts annotated as WD40 repeat family protein from sorghum and maize on Phytozome 12. The presence of the WD40 motif (PF00400) was verified in each protein sequence using Pfam. This resulted in 169 WD40 proteins in sorghum and 235 in maize. From these, 113 and 176 respectively showed only the WD40 motif. Next, we performed a BLASTN search on the sugarcane assembled transcriptome (TR7) (Santa Brigida et al., 2016) using the scaffold5050 to obtain the possible transcript from this region. We found one locus (Locus_26521) with zero e-value. The Pfam analysis of the predicted protein from the locus_26521 showed the presence of three domains: WD-40 (PF0400), BEACH (PF02138) and PH_BEACH (PF14844). These domains were characterized in the subfamily C of WD40 proteins in *Setaria italica* (Mishra et al., 2014). Considering the subfamily C of WD40 in *S. italica*, we selected all sorghum and maize proteins containing WD-40 and BEACH domains for a phylogenetic analysis. A phylogenetic tree was constructed with these WD40 proteins using the maximum-likelihood method (Fig. 4). Three different subgroups were found on the WD40 subfamily C. The proteins (Sobic.001G093800.1, GRMZM2G056645_T01, and Locus_26521_transcript_1) formed one subgroup, which confirmed the annotation of the scaffold5050 (Table 2).

**Figure 5:**
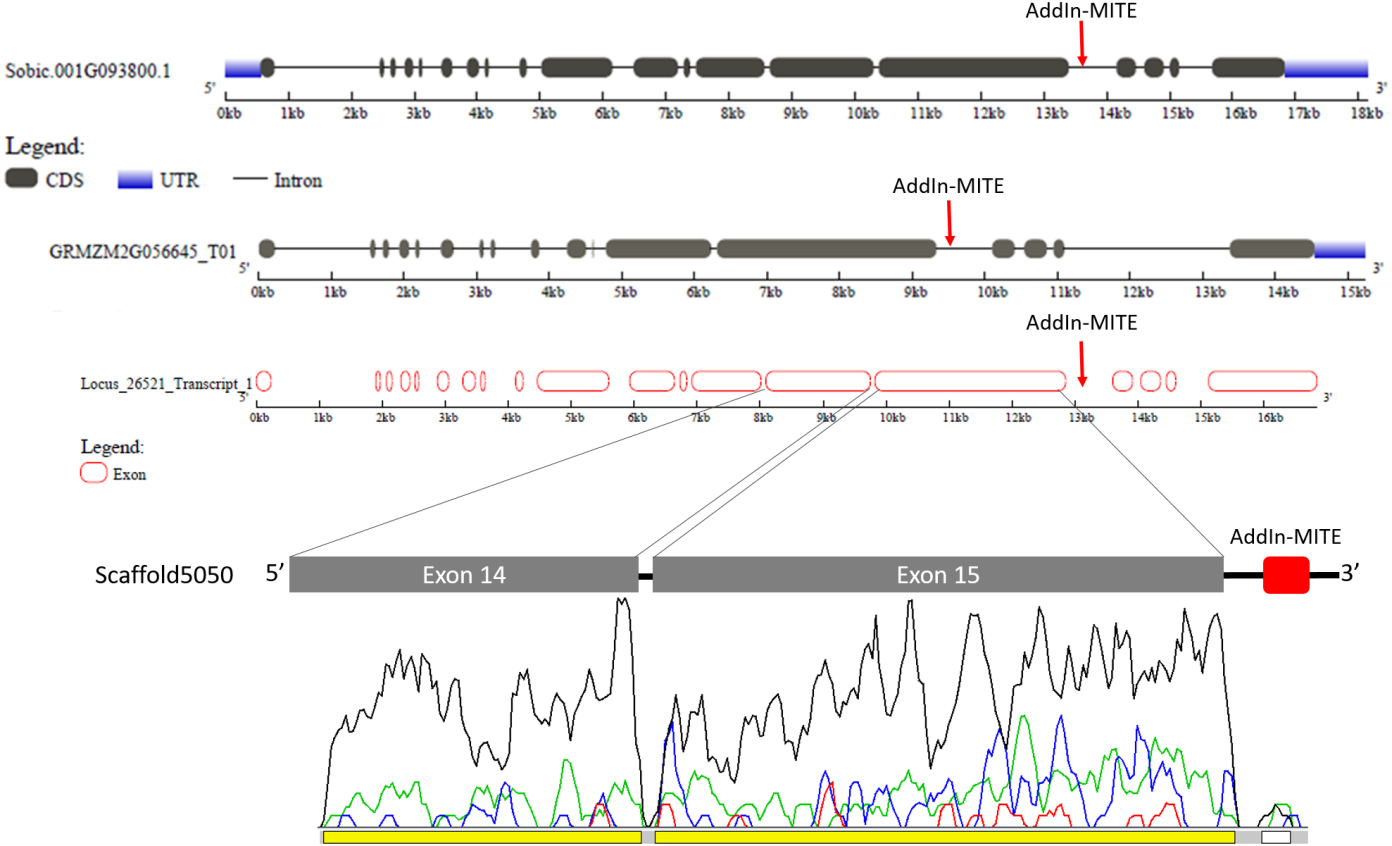
WD40 gene structure comparison between sorghum, maize, and sugarcane. The structure of WD40 genic regions from sorghum and maize are shown. The AddIn-MITE on these regions is highlighted with red arrows. The Locus_26251 from sugarcane and the Scaffold5050 containing the WD40 exons and the AddIn-MITE are also shown. The transcriptional evaluation of exon regions on the scaffolds containing the WD40 gene was performed with RNA-seq data. The color lines represent the coverage mapping of RNA-seq reads of four sugarcane control samples in the WD40 exons and AddIn-MITE. AddIn-MITE was marked with a white box and exons with yellow boxes.

The gene structure of Sobic.001G093800 and GRMZM2G056645 was compared with GSDS web tool (Fig. 5). The WD40 gene structure was similar between sorghum and maize. We also compared the Locus_26521_transcript_1 and Scaffold5050 predicted exons with those sorghum and maize genes (Fig. 5). A second annotation verification in WD40 exons regions at the scaffold5050 using RNA-seq reads was performed. The transcriptome libraries from sugarcane plants grown in control hydroponics and *in vitro* conditions were aligned against the scaffold5050 containing the WD40 exons and AddIn-MITE. The Locus_26251 assembled from RNA-seq data of sugarcane (Santa Brigida et al., 2016) and the peaks of transcription at the two exons regions (Fig. 4), confirmed the position of the WD40 exons on the sugarcane scaffold5050.

**Figure 6:**
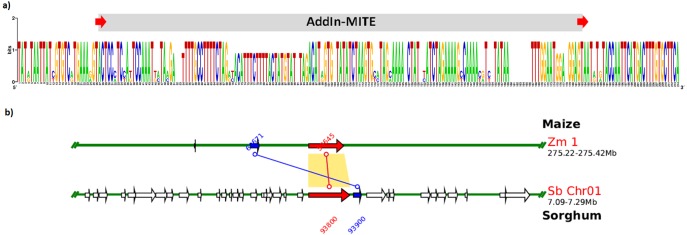
Sequence conservation and synteny analysis of WD40 genic region containing the AddIn-MITE. (A) The AddIn-MITE region and flanking sequences was extracted from sugarcane, sorghum and maize WD40 gene, aligned using MUSCLE and compared with WebLogo. (B) Synteny analysis was performed on Plant Genome Duplication Database (PGDD) using maize gene ID close related with sorghum and sugarcane. The arrows in red indicate the WD40 genes in sorghum and maize chromosomes. The number of the genes are also indicated in the figure with red and blue letters.

We also verified that AddIn-MITE was located close to WD40 exons in both sorghum and maize (Fig. 5). Then, we analyzed the flanking sequences and the orientation of AddIn-MITE. The orientation of AddIn-MITE was the same in the three species and their flanking sequences showed great conservation (Fig. 6A). The analysis of synteny on Plant Genome Duplication Database (PGDD) reveals a huge block on chromosome 1 of maize and sorghum, which include the WD40 genes of both species (Fig. 6B). These results suggest that the AddIn-MITE inserted close to WD40 exons before the divergence of sugarcane, sorghum, and maize.

### Profile of AddIn-MITE-related sRNAs on sugarcane WD40 gene

**Figure 7:**
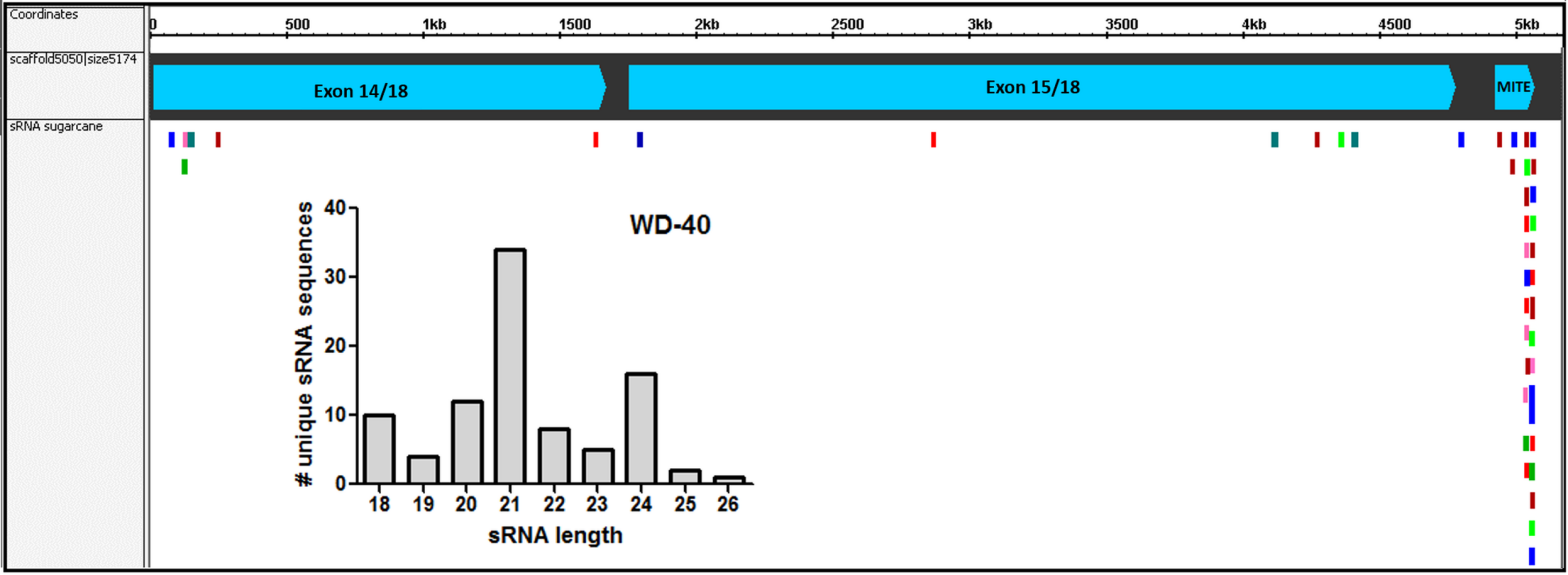
Small-RNAs derived from AddIn-MITE. The alignments of sRNAs from sugarcane leaves to WD40 genic region are shown. The alignments were performed with non-redundant siRNA and zero mismatches allowed. Bars graphs showed the distribution of sRNA by size that mapped at the gene sequence.

Since the first report by Llave and coworkers in Llave et al. (2002), the knowledge about the roles of sRNA regulating gene expression in plants has been expanded enormously. In recent years, the association between repetitive genomic regions and sRNA has been described. TE-derived sRNA has an important role in the feedback silencing of the active TEs (Ito, 2013). To evaluate if the AddIn-MITE located inside WD40 gene could generate MITE-derived sRNAs, we used sRNAs libraries constructed from sugarcane leaves and mapped them to the sugarcane genomic assembly (MF scaffolds) with zero mismatches. The sRNA mapping showed that on the scaffold5050 the majority of the mapped sequences are derived from the AddIn-MITE region (Fig. 7). The distribution of sRNA size showed that the sRNAs with 21-nt were the most abundant among the mapped sequences (Fig. 7). The WD40 genes containing the AddIn-MITE in sorghum and maize were also checked for the alignment of sRNAs from leaf libraries. The mapping on sorghum showed very few sRNAs on AddIn-MITE region, most of them with 21-nt in size (Fig. S3A). Curiously, the WD40 genes from maize did not show sRNAs mapped in the AddIn-MITE region with zero mismatches (Fig. S3B).

**Figure 8:**
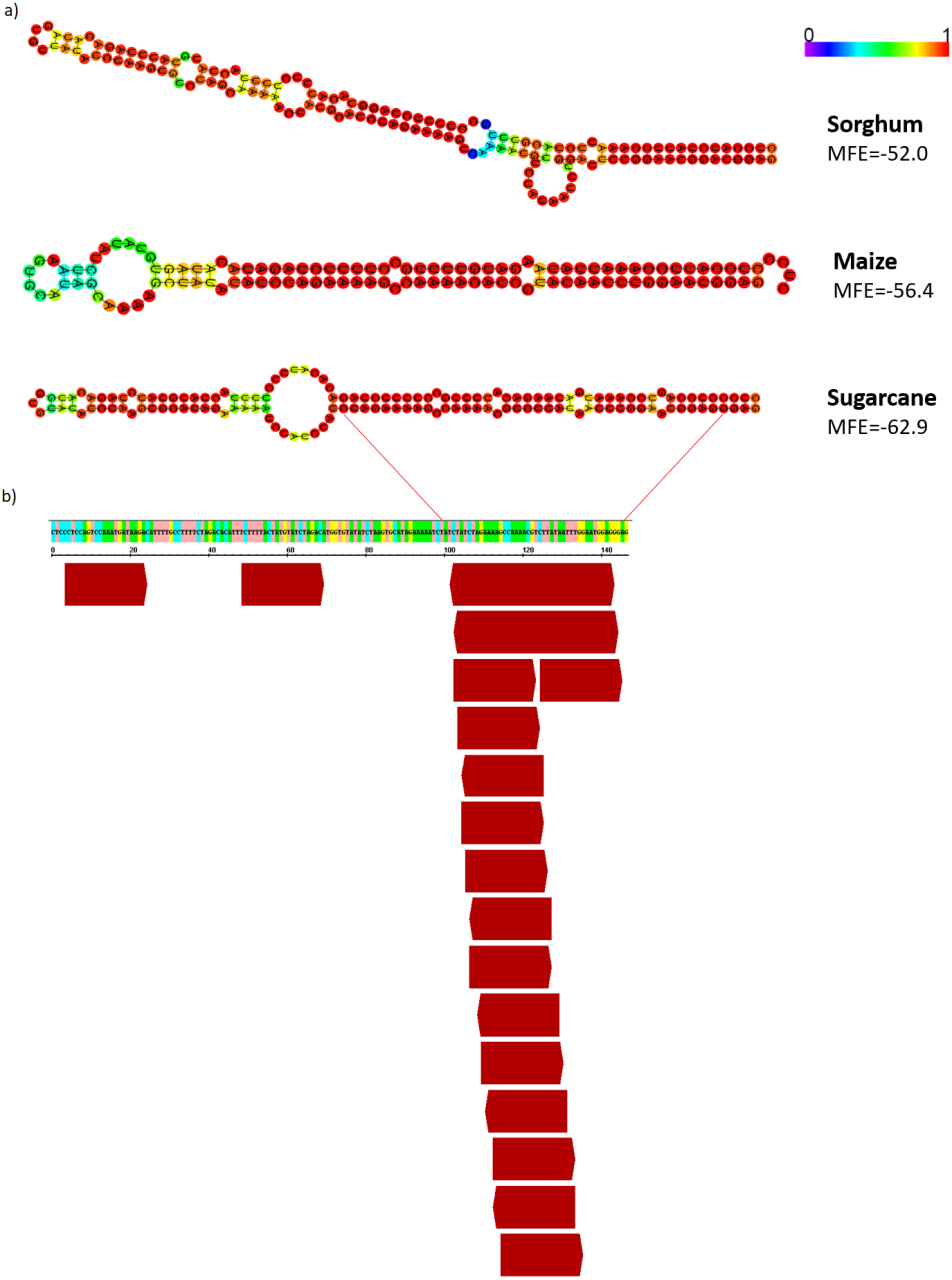
AddIn-MITE-derived hairpin structure predicted from sugarcane, sorghum and, maize on WD40 gene. (A) The hairpin structure was predicted from AddIn-MITE regions from grasses species. (B) The profile of 21-nt sRNA mapping on AddIn-MITE was also shown. sRNA library from sugarcane leaves (*Saccharum spp*. SP70-1143) was used to analyze the amounts of 21-nt sRNA alignment within AddIn-MITE region.

Since the majority of canonical plant miRNA has 21-nt in length and are produced from a hairpin precursor (Lee et al., 2015), the 21-nt sequences mapping on the sugarcane scaffold5050 could be the initial step for the emergence of a new miRNA precursor. To evaluate this, we compared the hairpin structure from each AddIn-MITE region of maize, sorghum, and sugarcane (Fig. 8A). The hairpin structure in the AddIn-MITE region was found on sugarcane WD40 gene and showed the lowest Minimum Free Energy (MFE = −62.9). The sugarcane AddIn-MITE on WD40 gene also showed a pattern of 21-nt small RNA mapping on its 3′ end (Fig. 8B), which would suggest that this region is a “proto-miRNA” locus in sugarcane. This seems to be corroborated by the profile of sRNAs mapped in the AddIn-MITE region on WD40 gene. Seventeen sRNAs from sugarcane plants subjected to pathogen infection and to salt stress mapped to AddIn-MITE (Fig. S4A). From these, nine have 21-nt in length (Fig. S4).

## Discussion

Recently, the well-known phenotype of color change of British peppered moth during Industrial Revolution was implicated to a TE insertion in the gene *cortex*, which increased the expression of the *cortex* transcript (Hof et al., 2016). This discovery highlighted the importance of TEs in the regulation of genes and consequent phenotypic changes. Interestingly, 85% of maize genome is composed of TEs (Schnable et al., 2009), and most of them must be silenced and inactivated to maintain the stability of the genome (Lisch, 2012). In bacterial artificial chromosome (BAC) assemblies of sugarcane, 49.4% of sequences are TEs, and among them, 3% were classified as MITEs (De Setta et al., 2014). Based on the structure of MITEs, novel MITEs can be identified through a search for sequences with TIRs and TSDs regions (Lu et al., 2012). MITE-Hunter has emerged as an important tool for *de novo* identification of MITEs (Han & Wessler, 2010). Here, we identified eight different MITEs within 145 sugarcane methyl-filtered genomic sequences using the MITE-Hunter pipeline and additional steps to select TEs with TSDs sequences. The MITEs’ sequences ranged from 106 to 155 bp with 2 to 4 bp of TSDs sequence, in agreement with characteristics of most of the MITEs, which are usually shorter than 500 bp in length and can have TSDs varying from 2 to more than 10 bp (Chen et al., 2014a). These MITEs were classified into two superfamilies’ previously identified in plant genomes (Bureau & Wessler, 1992; Bureau & Wessler, 1994). Interestingly, the MITE sugarcane_1_2650 superfamily was not identified (Table 1), suggesting that maybe this MITE could be sugarcane lineage specific. The number of MITEs varies among the plant genomes. For instance, in *Sorghum bicolor,* there are 275 MITE families with more than one thousand elements, while *Carica papaya* has only one MITE family with 538 elements (Chen et al., 2014b). Although the distribution of MITEs in the plant genomes can be highly variable, they are inserted predominantly in genic-rich regions (Zhang, Arbuckle & Wessler, 2000; Feschotte, Jiang & Wessler, 2002; Han, Qin & Wessler, 2013). In the rice cultivar Nipponbare, approximately 58% of the genes have MITE insertions in their introns or flanking regions (Lu et al., 2012). Here, we found that the majority of identified sugarcane MITEs is localized at intronic regions. Furthermore, we observed a close physical association between MITEs and genes related to plant development, hormone response, cell wall, stress response and epigenetic modification. These results suggest that MITEs can be associated with gene regulation and genome evolution of sugarcane.

The evolution of MITEs depends on factors that determine the MITE insertion, which can differ among MITE families (Zerjal et al., 2009). Thus, some MITE families could have been amplified recently or could be the result of ancient amplifications. A study showed conservation of 85% of the MITE loci in two rice genotypes, and that a larger proportion of MITE insertions is fixed in 25 rice cultivars of *O. sativa* (Chen et al., 2012). Although the MITE family *Gaijin*-like MITEs (mGing) are present in all analyzed rice cultivars, there are some individual insertions that are polymorphic among cultivars (Dong et al., 2012). Interestingly, the AddIn-MITE position nearby two genes, WD40 and CALS8, was conserved in all analyzed sugarcane cultivars and five wild *Saccharum* species, indicating that these insertions maybe be ancient and fixed in the *Saccharum* lineage. In our analysis, a sugarcane MITE (AddIn-MITE) was found to be also conserved in closely related species of Panicoideae, but not in *Oryza* and *Brachypodium* species, indicating that the MITE family emerged and was amplified after the divergence of Panicoideae and other grasses (Kellogg, 2001). Chen and collaborators (2012) also highlighted that the estimated amplification time of some MITEs families is more recent than the divergence time between *Oryza* and *Brachypodium*. Another study showed that insertion of one MITE in maize occurred after maize domestication because the MITE is not present in the teosinte accession, the wild ancestor of maize (Mao et al., 2015). Furthermore, this MITE was found in the promoter region of the ZmNAC111 gene only in drought-sensitive maize genotypes, and its insertion resulted in a reduced expression of this gene. Other studies also highlighted that MITEs may affect the expression of nearby genes via MITE-derived sRNA (Kuang et al., 2009; Lu et al., 2012). Due to the proximity of the AddIn-MITE with the WD40 sugarcane gene, it is possible that the MITE can affect the expression of the gene through the sRNA feedback regulation pathway. Accordingly, the majority of sRNA from sugarcane leaves mapped on WD40 are AddIn-MITE-derived sRNA. In rice, around one-quarter of rice sRNAs are generated by MITE sequences (Lu et al., 2012). Interestingly, very few or no one sRNA from sorghum and maize leaves mapped on AddIn-MITE regions on WD40 genes.

The evolutionary transition of siRNA-to-miRNA in MITEs regions has been reported for Arabidopsis and rice (Piriyapongsa & Jordan, 2008). These authors showed that MITEs could be expressed from intronic regions and form a miRNA-like hairpin structure from the pairing of TIR sequences. The evolution of miRNA precursors in plants is supposed to start with a stage where small RNAs are generated from inverted duplications by one or more DCL enzymes (Cuperus, Fahlgren & Carrington, 2011; Vazquez et al., 2008). MITEs can contributed with this initial stage, once they provide inverted repeats, which can be transcribed as hairpins resembling proto-miRNAs. In this initial stage, the generation of heterogeneous sRNAs populations from the same hairpin seems to be advantageous to settle down the regulation of the target to the precise processing of canonical miRNAs (Axtell, Westholm & Lai, 2011). The evolution of a proto-miRNA locus to a canonical hairpin is dependent of acquisition of DCL1 processing of sRNA species by drift mutations, selecting the sRNA within the fold-back sequence to produce a young MIR gene (Axtell, Westholm & Lai, 2011; Voinnet, 2009). Here, we have shown that in sugarcane AddIn-MITE region form a stem-loop structure with the lowest MFE compared to sorghum and maize and showed a preferential mapping of 21-nt sRNAs on AddIn-MITE 3′ end, suggesting that AddIn-MITE is a proto-miRNA locus, maybe evolving to be a young MIR gene. A previous study with rice has shown that an Stowaway-like MITE (sMITE) can generate 21- and 24-nt sRNAs and they can regulate the translation of Gdh2 gene (Shen et al., 2017). Since members of the WD40 family proteins have been correlated to biotic and abiotic stress responses (Huang et al., 2008; Lee et al., 2010; Kong et al., 2015; Miller, Chezem & Clay, 2015), we speculate that the sRNA derived from AddIn-MITE region could exert a role as WD40 regulator.

## Conclusion

In this study, we investigated the TE class II—MITEs—in sugarcane methyl-filtered genome assembly in sugarcane. The closest relationship of identified sugarcane MITEs with genes involved in different biological functions was found. One of those identified MITEs (AddIn-MITE) were abundant within the genes, especially in genes containing WD40 domains. A comparative analysis of AddIn-MITE in monocots’ genomes showed a great conservancy of this element in Panicoid. A more accurate analysis of sorghum and maize WD40 gene showed that similar to observed in sugarcane, both grasses have AddIn-MITE located at intronic regions, which possible inserted before the divergence of these species. Examination of the AddIn-MITE-WD40 in sugarcane wild species and cultivars, allowed us to obtain information on the organization and distribution of the AddIn-MITE in the *Saccharum* lineage. In addition, the analysis of the small RNA distribution patterns in the WD40 gene suggests that the AddIn-MITE region is arising as a proto-miRNA locus. Together, our data provide insights into the overall composition of MITEs in sugarcane, and improve the understanding of the relationship between MITEs, genes and small RNAs in Andropogoneae grasses.

##  Supplemental Information

10.7717/peerj.6080/supp-1 Supplemental Information 1 Supplementary dataset

10.7717/peerj.6080/supp-2 Supplemental Information 2 Supplementary figures

10.7717/peerj.6080/supp-3 Supplemental Information 3 Supplementary tables

10.7717/peerj.6080/supp-4 Supplemental Information 4 Full length gel blot of AddIn-MITE’s position nearby WD40 gene PCR reaction of the combination of primers (MITE/WD40) with gDNA from sugarcane wild species –*S. officinarum, S. spontaneum, S.robustum, S. sinense, S. barberi* - and cultivars –B4362, Uruguai.

10.7717/peerj.6080/supp-5 Supplemental Information 5 Full length gel blot of AddIn-MITE’s position nearby WD40 gene PCR reaction of the combination of primers (MITE/WD40) with gDNA from the cultivars Branca Durona, RB72454 and RB867515.

10.7717/peerj.6080/supp-6 Supplemental Information 6 Full length gel blot of AddIn-MITE’s position nearby gene PCR reaction with gDNA from the SP70-1143 sugarcane cultivar showed the distance between the AddIn-MITE and the WD40 exon region. M-100bp ladder was used to confirm the length of the PCR products.

## Additional Information and Declarations

### Data Availability

The following information was supplied regarding data availability: The research in this article did not generate any raw data. The MITEs identification sequences are available as Supplemental Material.
