# Supplementary material for: A miniature inverted-repeat transposable element, AddIn-MITE, located inside a WD40 gene is conserved in Andropogoneae grasses
Source: PeerJ. 2019 Jan 11;7:e6080. doi: 10.7717/peerj.6080 (PMC6331000; doi:10.7717/peerj.6080)
Supplement: Supplemental Information 2 [file peerj-07-6080-s002.pdf]

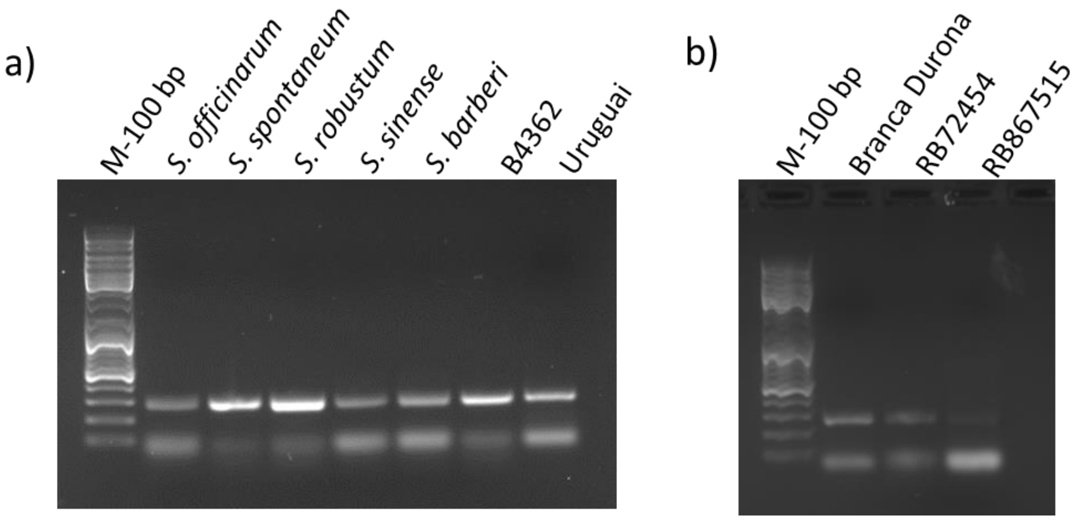

Figure S1 – AddIn-MITE's position nearby WD40 gene. PCR reaction of the combination of primers (MITE/WD40) with gDNA from sugarcane wild species – *S. officinarum, S. spontaneum, S.robustum, S. sinense, S. barberi* (a) - and cultivars – B4362, Uruguai (a) and Branca Durona, RB72454, RB867515 (b). The figure showed the amplified region between the MITE and the WD40 exon. As shown on Figure 3, the expected size is 282bp. M-100 bp ladder was used to confirm the length of the PCR products.

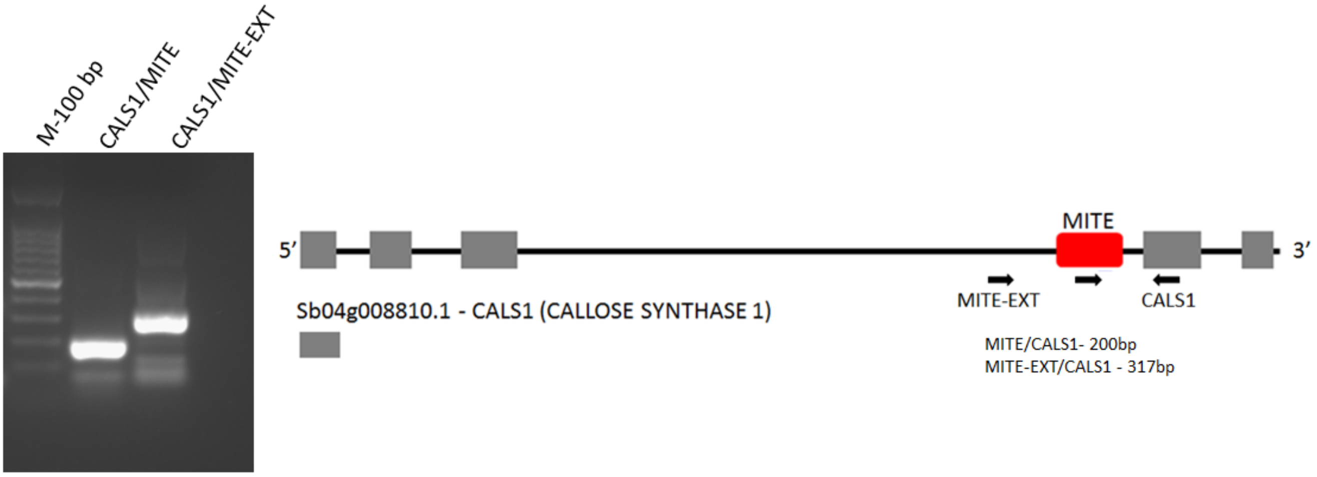

Figure S2 – AddIn-MITE's position nearby CALS1 gene. PCR reaction with gDNA from SP70-1143 sugarcane cultivar showed the distance among the MITE and the CDS region. The scheme illustrated how the primers were designed (black arrows). M-100 bp ladder was used to confirm the length of the PCR products.

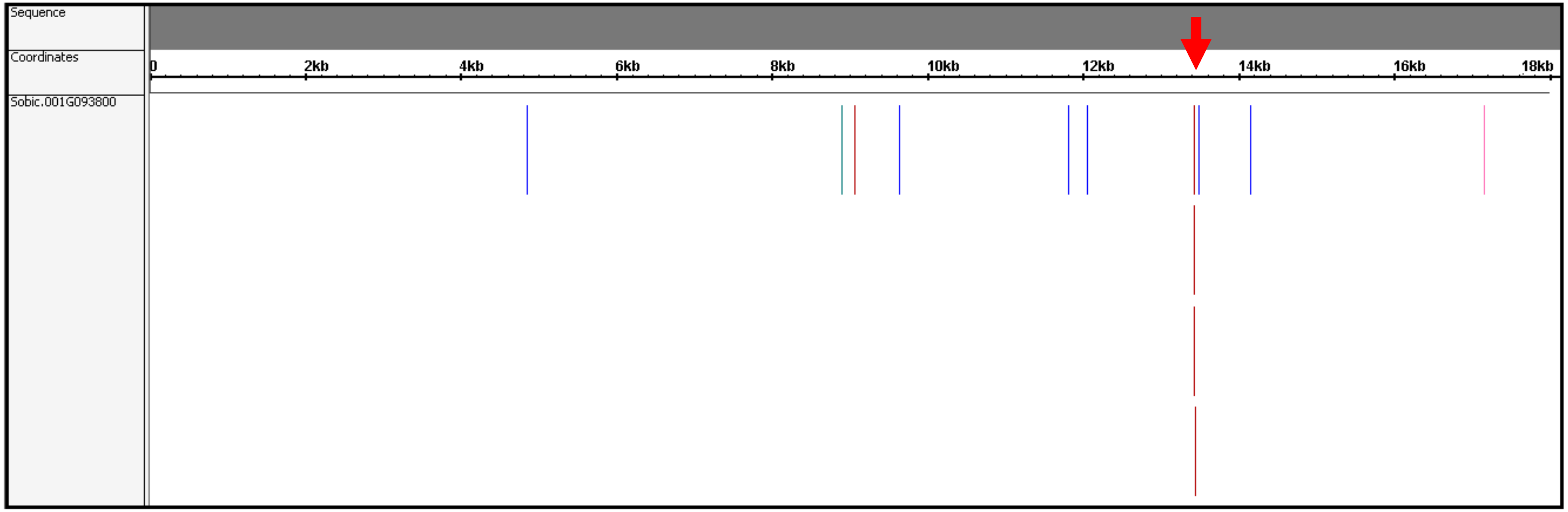

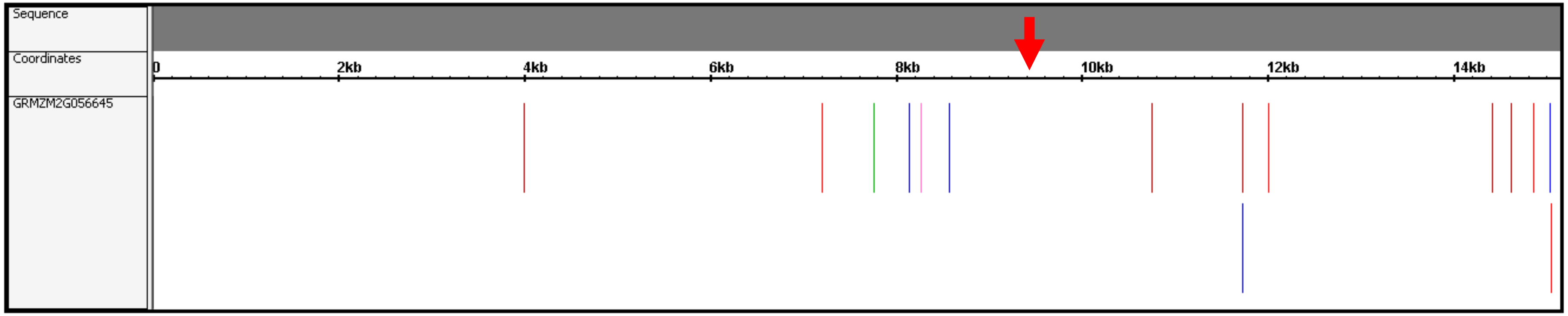

Figure S3- Small-RNAs mapping on WD40 gene containing AddIn-MITE. The alignments of sRNAs from sorghum (above panel) and maize (below panel) leaves to WD40 genic region are shown. The alignments were performed with non-redundant siRNA and zero mismatches allowed. Bars graphs showed the distribution of sRNA by size that mapped at the gene sequence. The red arrow indicates the AddIn-MITE position.

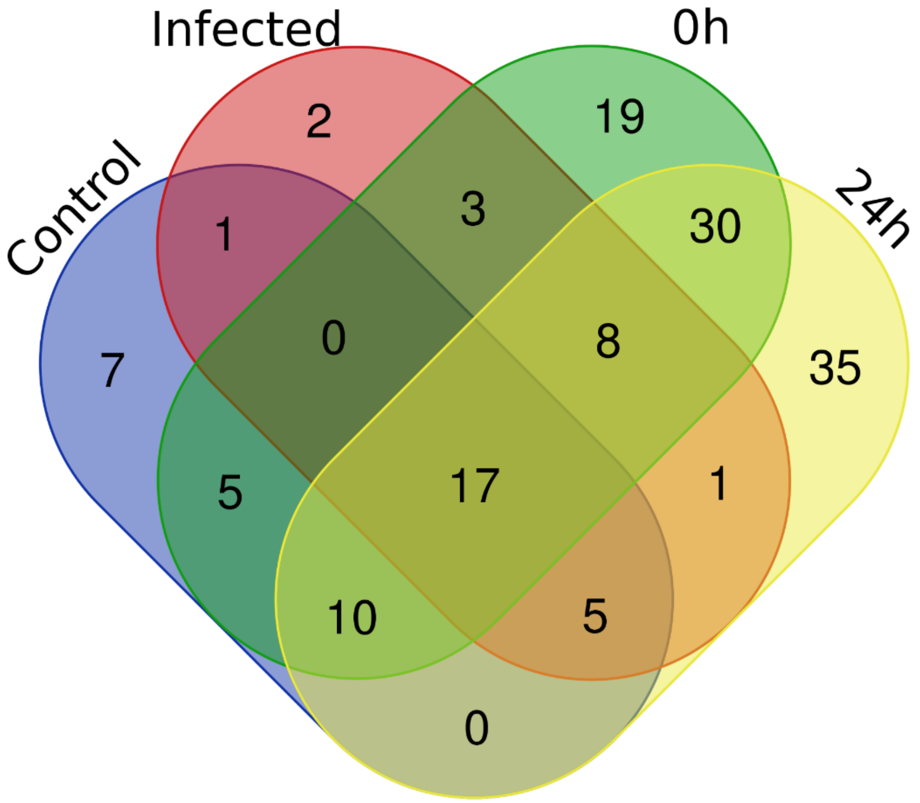

Figure S4 – Venn diagram of AddIn-MITE-derived sRNA at neighbor gene (WD40) in sugarcane. Data of sRNA libraries from sugarcane after 0 and 24h of salt stress and subjected to pathogen infection were used. The sRNA reads matching with the AddIn-MITE was compared between libraries.
