# Supplementary figures and images for: A miniature inverted-repeat transposable element, AddIn-MITE, located inside a WD40 gene is conserved in Andropogoneae grasses
Source: PeerJ. 2019 Jan 11;7:e6080. doi: 10.7717/peerj.6080 (PMC6331000; doi:10.7717/peerj.6080)

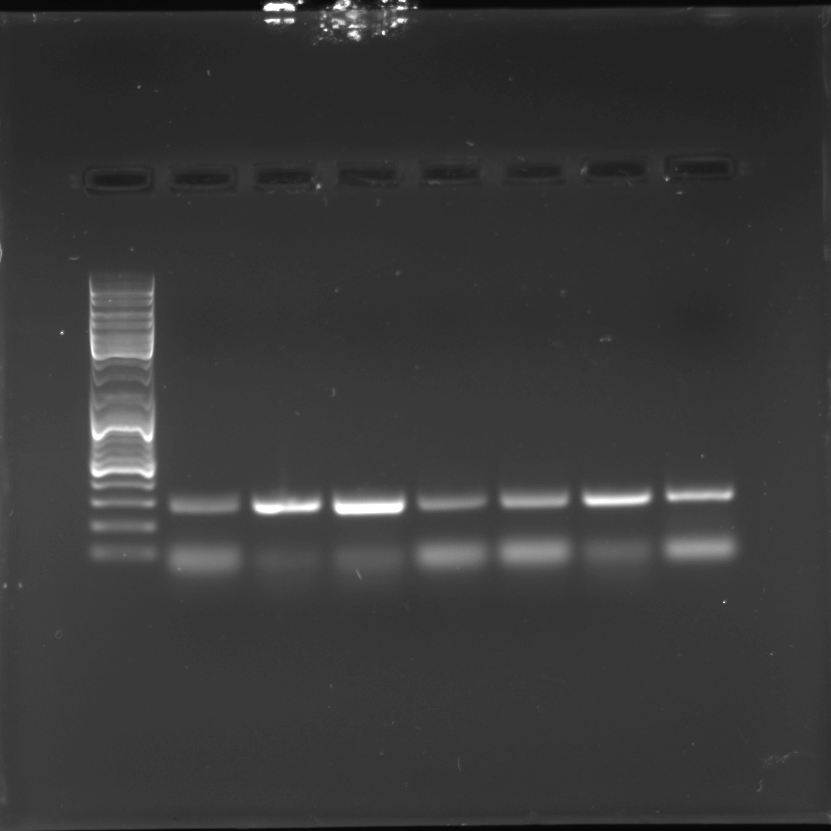

Supplement: Supplemental Information 4 — PCR reaction of the combination of primers (MITE/WD40) with gDNA from sugarcane wild species –S. officinarum, S. spontaneum, S.robustum, S. sinense, S. barberi - and cultivars –B4362, Uruguai. [file peerj-07-6080-s004.png]

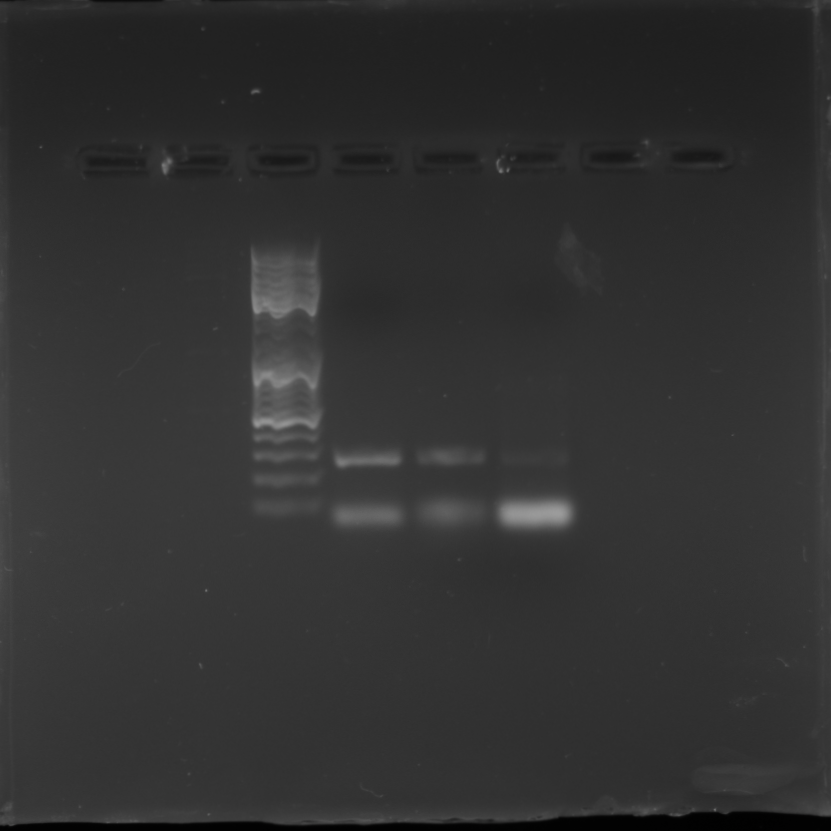

Supplement: Supplemental Information 5 — PCR reaction of the combination of primers (MITE/WD40) with gDNA from the cultivars Branca Durona, RB72454 and RB867515. [file peerj-07-6080-s005.png]

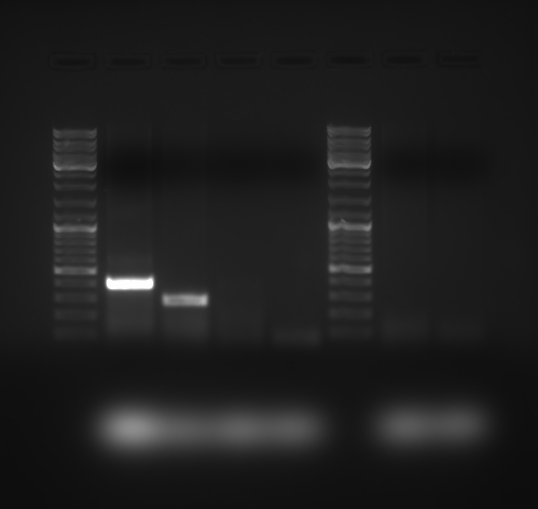

Supplement: Supplemental Information 6 — PCR reaction with gDNA from the SP70-1143 sugarcane cultivar showed the distance between the AddIn-MITE and the WD40 exon region. M-100bp ladder was used to confirm the length of the PCR products. [file peerj-07-6080-s006.png]
